# The Best Summer Beverage

**Published:** 1880-08

**Authors:** 


**The Best Summer Beverage**, is cold water, ice cold, if you please, but by all means grasp the glass by the hand, take a swallow at a time, remove the glass from the lips for a few seconds then take another swallow; in this way it will be found that the thirst will be thoroughly satiated before half the water has been taken, whereas if it had been swallowed continously, the whole contents would not have satisfied the thirst.

As many persons have dropped dead from drinking greedily of water not ice cold, when very much heated and fatigued, this precaution may save many a life.

Butter-milk is another admirable summer beverage. Its acidity cools the system and acts slightly on the liver, and thus promotes the passing of the bile and other impurities from the body.

